# Study on polymorphisms in CHRNA5/CHRNA3/CHRNB4 gene cluster and the associated with the risk of non-small cell lung cancer

**DOI:** 10.18632/oncotarget.23459

**Published:** 2017-12-20

**Authors:** Yiting Sun, Jiaye Li, Chang Zheng, Baosen Zhou

**Affiliations:** ^1^ Department of Clinical Epidemiology, First Affiliated Hospital, China Medical University, Shenyang, China; ^2^ Key Laboratory of Cancer Etiology and Prevention, China Medical University, Liaoning Provincial Department of Education, Liaoning, China; ^3^ First Clinical College, China Medical University, Shenyang, China

**Keywords:** CHRNA5/CHRNA3/CHRNB4, single nucleotide polymorphism, non-small cell lung cancer, smoking, interaction

## Abstract

CHRNA5/CHRNA3/CHRNB4 gene cluster is located on chromosome 15q25.1 and was reported to be associated with risk of lung cancer. So far, the effect of three single nucleotide polymorphisms rs6495309, rs8040868, rs1948 in this gene cluster was unclear about lung cancer risk. The aim of the present study was to evaluate the associations of rs6495309, rs8040868, rs1948 polymorphism, smoking exposure and the interaction with non-small cell lung cancer risk in Chinese population. In this hospital-based case-control study, 306 lung cancer patients and 306 cancer-free controls were interviewed to collect demographic data and exposure status of smoking, and then donate 2ml venous blood which was used to be genotyped by Taqman allelic discrimination method. Our study found that subjects carrying rs1948 CT genotype stated to be a risk factor in Chinese Han population (adjusted OR = 1.594, 95% CI = 1.066-2.383, *P* = 0.023) and in non-smoking population (adjusted OR = 1.896, 95%CI = 1.069–3.362, *P* = 0.029). rs8040868 CC genotype indicated a higher risk for lung cancer in non-smokers in a recessive model (adjusted OR = 2.496, 95%CI = 1.044–5.965, *P* = 0.040) and in age-based stratified analysis (age <= 60, adjusted OR = 4.213, 95%CI = 1.062-16.708, *P* = 0.041). All smoking interaction were positive in the multiplicative interaction of the SNPs and smoking status (-/+) compared with recessive model. Overall, these finding suggested that rs1948(C > T) and rs8040868(T > C) could be meaningful as genetic markers for lung cancer risk in Chinese Han population.

## INTRODUCTION

Lung cancer is the leading cause of cancer-related death and the most common cancer worldwide [1, 2]. The incidence and mortality rate of lung cancer is increasing nowadays. It is reported that genetic factors contribute to the risk for smoking initiation [3], first smoking experiences, cigarettes smoked per day (CPD), nicotine dependence, and persistent smoking which are risk factors of lung cancer [4]. Genome-wide association studies (GWAS) have highlighted the prevailing view that smoking quantity and frequency as a proxy for nicotine dependence is most associated with single nucleotide polymorphisms (SNPs) at the CHRNA5/CHRNA3/CHRNB4 gene cluster [5]. Activation of nAChRs suppresses cell apoptosis and stimulates the growth of lung cancer cells, which indicates that nicotine can lead to lung cancer development by acting as tumor promoters that results in the outgrowth of cells with genetic damage [6].

Neuronal nicotinic acetylcholine receptor (nAChR) gene cluster (CHRNA5/CHRNA3/CHRNB4) on chromosome 15q25.1 belongs to the ligand-gated ion channel superfamily which widely distributing in the brain that binds the ligands acetylcholine and nicotine [7], which mediates fast cholinergic synaptic transmission, involving in activation of downstream signaling networks that promote cell proliferation, migration, invasion, and angiogenesis [8]. nAChRs also have been considered to be associated with nicotine dependence and smoking behaviors as well as chronic obstructive pulmonary disease (COPD) and lung cancer risk [9].

We evaluated association of several SNPs in the CHRNA5/A3/B4 gene encoding nAChR subunit with lung cancer measuring frequencies of minor alleles and genotype distribution [10]. CHRNA3/B4 intergenic SNPs rs6495309, located on the promoter, has been associated with nicotine dependence, COPD and risk for lung cancer. SNP rs1948 locates in the CHRNB4 3’-untranslated region (UTR), a region known to participate in the stability/instability of mRNA and has been associated with early age of tobacco initiation [11]. Early initiation for tobacco use is a strong predictor of future nicotine dependence and lung cancer risk [12]. An analysis for detection of expression quantitative trait loci revealed that CHRNA3 rs8040868-C allele was found to be closely associated with a decreased expression of the nearby gene CHRNA5 cholinergic receptor in lung tissue and conferred a risk for COPD, in which CHRNA3 and CHRNA5 are located in a tail-to-tail configuration on opposite DNA strands and share some of their 3′-untranslated region [13].

A report suggested that epigenetic deregulation of nAChR genes are strongly associated with genetic lung cancer susceptibility variants and a functional impact on tumorigenic potential [14] and have an important role in mediating the effect of nicotine on the dopaminergic pathway and dopamine release from limbic brain areas. Studies have shown that CHRNA3 associated with smoking addiction through the high expression in the key regions of brain [15]. Notably, rs6495309 in the promoter of the CHRNA3 gene was reported to affect the binding ability of the transcriptional factor Oct-1 that has been shown to repress gene transcription, resulting in alteration of CHRNA3 RNA expression, impacting the ability to enter into apoptosis, thereby influencing lung cancer risk [16, 17].

The most consistent overall DNA methylation difference between tumor and adjacent normal tissue on 15q25 was tumor hypomethylation in the promoter region of CHRNB4, which resulted in overexpression of the transcript in tumors [18]. It also reported hypermethylation in CHRNA3 and telomerase reverse transcriptase (TERT) with significant expression changes in previous studies and methylation events frequently lead to gene silencing of potential tumor suppressor genes. Also, there are reports demonstrate that SNP rs1948 alters luciferase expression when sequences carrying this genetic variant were cloned downstream of the luciferase gene, a recreation of rs1948 in relation to the CHRNB4 3’-UTR gene [19]. Additionally, in mice studies, deletion of the a5 or overexpression of the b4 of nAChR subunits has been shown to reduced sensitivity to nicotine-induced seizures [20].

Nicotine and nicotine-derived nitrosamines in cigarette smokers triggers the nAChR in lung cells to release protease and oxidants that are associated with the pathogenesis of lung cancer [5]. Based on SNPs rs1948, rs6495309, rs8040868 reside in inclusive of the 3′ UTR of CHRNB4, a noncoding region upstream of CHRNA3 promoter and exon2 respectively, we did a further research to determine whether the three SNPs differentially affect lung cancer risk with smoking exposure.

## RESULTS

The demographic details of the characteristics of patients and cancer-free control subjects involved in the study are shown in Table 1. There were 306 lung cancer patients and 306 controls in the present study. Among the lung cancer patients, there were 140 adenocarcinoma, 123 squamous cell carcinoma and 43 other types in pathologic type of lung cancer. The minor allele frequency (MAF) of rs6495309, rs8040868, rs1948 is 0.500,0.695,0.450 respectively. (https://www.ncbi.nlm.nih.gov/projects/SNP/snp_ref.cgi?rs=6495309) (https://www.ncbi.nlm.nih.gov/projects/SNP/snp_ref.cgi?rs=8040868) (https://www.ncbi.nlm.nih.gov/projects/SNP/snp_ref.cgi?rs=1948) The mean age for case group and control group were 62.2 ± 8.9 and 58.2 ± 13.9 respectively. The result of χ^2^ test showed that there is no statistical difference in histological type between case and control group, which suggested that frequency-matching on histological type was suitable in our study (*P* > 0.05). All the differences were controlled in the later multivariate analyses. For all SNPs, the distribution of genotypes among the control subjects was in accordance with Hardy–Weinberg equilibrium, which suggested that our control group has an appropriate representativeness for the studying population.

**Table 1:** Basic characteristics of the study population

Characteristics	Case-control study		
	Cases (n = 306)	Controls (n = 306)	*P* value
Age (year) (mean±SD)	62.2±8.9	58.2±13.9	<0.001^a^
Gender			<0.001^b^
Male (%)	224(73.2)	149(48.7)	
Female (%)	82(26.8)	157(51.3)	
Smoking status			0.034^b^
Ever-smoker(%)	199(65.0)	87(28.4)	
Nonsmoker(%)	107(35.0)	219(71.6)	
Family history of cancer	298(97.4)	306(100.0)	<0.001^b^
Histological type			0.285^b^
Squamous-cell carcinoma	123(40.2)	—	
Adenocarcinoma	140(45.8)	—	
others	43(14.0)	—	

^a^*P* value was calculated by the t test.

^b^*P* value was calculated by the χ^2^ test.

Abbreviations: SD standard deviation.

We respectively studied associations between three SNPs and lung cancer risk in lung cancer patients and control subjects. Data is listed in Table 2. Of the SNP, the frequency of the heterozygous rs1948 CT genotype was 55.1 % in the case group and lower (49.7%) in the reference group. Among the three SNPs at 15q25, subjects carrying rs1948 CT genotype were significantly associated with an increased risk of lung cancer (adjusted OR = 1.594, 95% CI = 1.066-2.383, *P* = 0.023) compared to the subjects carrying homozygous CC genotype. In rs6495309 and rs8040868, no significant difference was found between the distributions of genotypes in two groups, which may due to the relatively small sample size and the results need to be further verified with a large sample population. We also analyzed the relationship of the three SNPs and the survival time but no significance result was found (Supplementary Table 3), which may need more cases to do further research.

**Table 2:** Genotypes of the single-nucleotide polymorphisms rs6495309, rs8040868, rs1948 in lung cancer patients and control subjects and their association with the risk of lung cancer

Genotype	Cases(%)	Controls(%)	OR(95% CI)	*P* value	OR_adj._(95% CI)	*P*_adj._
rs6495309						
C	333(54.4)	313(51.1)	Ref		Ref	
T	279(45.6)	299(48.9)	0.877(0.701-1.098)	0.252	0.892(0.706-1.128)	0.339
CC	90(29.4)	78(25.5)	Ref	0.500	Ref	0.613
CT	153(50.0)	157(51.3)	0.845(0.580-1.230)	0.379	0.924(0.624-1.367)	0.691
TT	63(20.6)	71(23.2)	0.769(0.488-1.212)	0.258	0.789(0.491-1.268)	0.328
CC+CT	243(79.4)	235(76.8)	Ref		Ref	
TT	63(20.6)	71(23.2)	0.858(0.585-1.259)	0.434	0.830(0.556-1.242)	0.365
rs8040868						
T	398(65.0)	432(71.3)	Ref		Ref	
C	214(35.0)	174(28.7)	1.335(1.048-1.700)	0.019^*^	1.267(0.985-1.630)	0.065
TT	125(40.4)	147(48.5)	Ref	0.041	Ref	0.147
TC	148(48.8)	138(45.5)	1.261(0.904-1.759)	0.172	1.238(0.875-1.750)	0.227
CC	33(10.8)	18(05.9)	2.156(1.158-4.015)	0.015^*^	1.813(0.954-3.446)	0.069
TT+TC	273(89.2)	285(94.1)	Ref		Ref	
CC	33(10.8)	18(05.9)	1.914(1.053-3.480)	0.033^*^	1.623(0.876-3.008)	0.124
rs1948						
C	303(50.0)	333(54.8)	Ref		Ref	
T	303(50.0)	275(45.2)	1.211(0.966-1.517)	0.096	1.229(0.971-1.556)	0.086
CC	68(22.4)	91(29.9)	Ref	0.112	Ref	0.068
CT	167(55.1)	151(49.7)	1.480(1.009-2.172)	0.045^*^	1.594(1.066-2.383)	0.023^*^
TT	68(22.4)	62(20.4)	1.468(0.921-2.339)	0.107	1.506(0.926-2.449)	0.099
CC+CT	235(77.6)	242(79.6)	Ref		Ref	
TT	68(22.4)	62(20.4)	1.129(0.766-1.665)	0.539	1.104(0.737-1.653)	0.632

^*^P<0.05.

In non-smoking population (Table 3), we found the rs1948 CT genotype carriers had great effects on increasing the risk for lung cancer when compared to homozygous wild type CC in non-smokers (adjusted OR = 1.896, 95%CI = 1.069–3.362, *P* = 0.029). Under a recessive model, rs8040868 had a relationship with a significantly increased risk of lung cancer for the variant allele (adjusted OR = 2.496, 95%CI = 1.044–5.965, *P* = 0.040).

**Table 3:** Genotype distribution and lung cancer risk in smokers and non-smokers

	Genotype	Cases(%)	Controls(%)	OR(95% CI)	*P* value	OR_adj._(95% CI)	*P*_adj._
Smokers	rs6495309						
	CC	60(32.1)	24(27.6)	Ref	0.752	Ref	0.612
	CT	88(47.1)	44(50.6)	0.800(0.441-1.452)	0.463	0.770(0.421-1.408)	0.396
	TT	39(20.9)	19(21.8)	0.821(0.398-1.694)	0.594	0.721(0.344-1.508)	0.384
	CC+CT	148(79.1)	68(78.2)	Ref		Ref	
	TT	39(20.9)	19(21.8)	0.943(0.508-1.757)	0.853	0.845(0.447-1.596)	0.604
	rs8040868						
	TT	70(37.4)	41(47.1)	Ref	0.316	Ref	0.262
	TC	97(51.9)	38(43.7)	1.495(0.873-2.560)	0.143	1.540(0.890-2.665)	0.123
	CC	20(10.7)	8(9.2)	1.464(0.592-3.623)	0.409	1.595(0.641-3.973)	0.316
	TT+TC	167(89.3)	79(90.8)	Ref		Ref	
	CC	20(10.7)	8(9.2)	1.183(0.499-2.802)	0.703	1.265(0.532-3.007)	0.595
	rs1948						
	CC	42(22.7)	24(27.6)	Ref	0.408	Ref	0.284
	CT	94(50.8)	46(52.9)	1.168(0.233-2.165)	0.62	1.241(0.661-2.327)	0.501
	TT	49(26.5)	17(19.5)	1.647(0.781-3.647)	0.19	1.834(0.859-3.912)	0.117
	CC+CT	136(73.5)	70(80.5)	Ref		Ref	
	TT	49(26.5)	17(19.5)	1.484(0.796-2.765)	0.214	1.584(0.845-2.968)	0.151
Non-smokers	rs6495309						
	CC	25(23.4)	54(24.7)	Ref	0.726	Ref	0.642
	CT	60(56.1)	113(51.6)	1.147(0.650-2.024)	0.636	1.174(0.660-2.089)	0.585
	TT	22(20.6)	52(23.7)	0.914(0.459-1.818)	0.797	0.894(0.445-1.796)	0.752
	CC+CT	85(79.4)	167(76.3)	Ref		Ref	
	TT	22(20.6)	52(23.7)	0.831(0.473-1.459)	0.52	0.800(0.451-1.418)	0.445
	rs8040868						
	TT	51(47.7)	106(49.1)	Ref	0.053	Ref	0.111
	TC	43(40.2)	100(46.3)	0.894(0.548-1.458)	0.653	0.900(0.574-1.478)	0.676
	CC	13(12.1)	10(4.6)	2.702(1.110-6.576)	0.029^*^	2.375(0.964-5.849)	0.060
	TT+TC	94(87.9)	206(95.4)	Ref		Ref	
	CC	13(12.1)	10(4.6)	2.849(1.206-6.731)	0.017^*^	2.496(1.044-5.965)	0.040^*^
	rs1948						
	CC	24(22.6)	67(30.9)	Ref	0.093	Ref	0.055
	CT	65(61.3)	105(48.4)	1.728(0.988-3.024)	0.055	1.896(1.069-3.362)	0.029^*^
	TT	17(16.0)	45(20.7)	1.055(0.510-2.182)	0.886	1.133(0.540-2.380)	0.741
	CC+CT	89(84.0)	172(79.3)	Ref		Ref	
	TT	17(16.0)	45(20.7)	0.730(0.395-1.349)	0.315	0.737(0.395-1.376)	0.339

^*^P<0.05.

We also performed a stratified analysis of the associations between three SNPs and lung cancer risk which was stratified by age (Supplementary Table 1). We found that in rs8040868 (age <= 60), CC genotype indicates a higher risk for lung cancer (adjusted OR = 4.213, 95%CI = 1.062-16.708, *P* = 0.041). Also, under a recessive model, rs8040868 was associated with a significantly increased risk of lung cancer for the variant allele (adjusted OR = 4.247, 95%CI = 1.101-16.380, *P* = 0.036).

In pathology-based stratified analysis (Supplementary Table 2), we analyzed the relationship of the three SNPs and lung adenocarcinoma as well as squamous cell carcinoma, but no significance result was found, which may need more cases to do further research.

To determine the direct joint effect of smoking and the three SNPs, we performed a logistic regression (Table 4) to estimate the multiplicative interaction of the SNPs and smoking status (-/+) compared with recessive model and no exposure as reference. We can find that all smoking interaction were positive.

**Table 4:** Interaction of three SNPs and smoking exposure on lung cancer risk

Smoking	Genotype	Cases	Controls	OR(95% CI)	*P* value	OR_adj._(95% CI)	*P*_adj._
	rs6495309						
−	CC+CT^*^non smoke	85	167	Ref	Ref	Ref	Ref
−	TT^*^non smoke	22	52	0.847(0.508-1.411)	0.523	0.890(0.528-1.500)	0.661
+	CC+CT^*^smoke	148	68	3.336(1.651-6.739)	0.001^*^	2.918(1.389-6.126)	0.005^*^
+	TT^*^smoke	39	19	3.537(2.091-5.983)	0.000^*^	3.201(1.787-5.733)	0.000^*^
	rs8040868						
−	TT+TC^*^non smoke	94	206	Ref	Ref	Ref	Ref
−	CC^*^non smoke	13	10	2.526(1.072-5.953)	0.034^*^	2.257(0.949-5.369)	0.066
+	TT+TC^*^smoke	167	79	4.108(2.879-5.863)	0.000^*^	3.594(2.295-5.629)	0.000^*^
+	CC^*^smoke	20	8	4.858(2.071-11.398)	0.000^*^	4.146(1.677-10.252)	0.002^*^
	rs1948						
−	CC+CT^*^non smoke	89	172	Ref	Ref	Ref	Ref
−	TT^*^non smoke	17	45	0.734(0.406-1.324)	0.304	0.735(0.403-1.339)	0.314
+	CC+CT^*^smoke	136	70	3.375(2.309-4.935)	0.000^*^	2.920(1.841-4.632)	0.000^*^
+	TT^*^smoke	49	17	5.008(2.736-9.166)	0.000^*^	4.258(2.183-8.306)	0.000^*^

^*^P<0.05.

## DISCUSSION

Lung cancer is one of the most common malignancy and major cause of death from cancer [21, 22]. Epidemiological data points out that the morbidity of lung cancer has been increasing in Chinese population every year. There is a tight connection between the occurrence of lung cancer and lots of environmental factors [23, 24], such as smoking behaviors, air pollution, occupation and so on [25, 26]. Besides, genetic factor is also an important factor which have great effects on lung cancer [27]. Recent years, many scientists have worked on studying the association between gene polymorphism, such as CHRNA5-CHRNA3-CHRNB4 polymorphisms, and lung caner susceptibility [28, 29]. However, not all results can be convinced because of several factors such as different races and insufficient cases [10]. Single nucleotide polymorphism is the most common pattern of human genetic variation. Some research indicates that some SNPs can influence the risk of cancer by affecting the expression and activity of enzyme [30–32], and people realize that SNP can be a potential biomarker of many varieties of cancers including lung cancer gradually [33–36]. Nowadays, there is a huge spectrum of opinion regarding to the etiology of lung cancer, but the pathogenesis of lung cancer has not been elucidated clearly. Some studies have demonstrated that the cluster of human neuronal nicotinic receptor gene CHRNA5-CHRNA3-CHRNB4 (15q25.1) is related to drug-related behaviors and the development of lung cancer. The interaction between SNP rs6495309 and nicotine dependence as well as SNP rs1948 and early use of drugs including tobacco and alcohol have an influence on the risk of lung cancer [1]. We chose and studied three SNPs based on the Genome-wide association study of our supervisor’s project team and focused on the association between CHRNA5-CHRNA3-CHRNB4 single nucleotide polymorphism rs6495309, rs1948 and rs8040868 and the risk of lung cancer in population, smokers and nonsmokers. In addition, we tried to dig out the influence of SNPs on lung cancer in different age groups, the interactive effects on lung cancer between these SNPs and smoking behaviors, and whether there is a relationship between SNPs and the prognosis of lung carcinoma. This study proves that genetic factors play an important role in the risk of lung cancer.

In resent research, we selected SNP rs6495309, rs1948 and rs8040868 in the cluster of genes CHRNA5-CHRNA3-CHRNB4 located on 15q25.1 and divided the genotype in Chinese Han population. The study suggests that SNP rs1948 (C > T) is significantly related to the risk of lung cancer, and the CT genotype has positive effects on it in not only Chinese Han population but non-smokers. In SNP rs8060868 (T > C), the allele C is a risk factor, for the reason that individuals with CC genotype contributes to a higher risk of lung cancer in non-smoking population and subjects (age <= 60). However, SNP rs6495309 (C > T) has less effect on the occurrence of lung cancer in population classified from different aspects, while the interaction between SNP rs6495309 and smoking behaviors can increase the risk of lung cancer.

We also found several previous surveys regarding to SNP rs6495309. Wu et al. found that SNP rs6495309 and rs2036524 located on 15q25 have a significant association with lung cancer in not only smokers and nonsmokers but also patients with squamous cell carcinoma and adenocarcinoma in Chinese Han population [6]. To verity the results of Wu et al, Bae et al. picked 1094 lung cancer patients and 1100 healthy controls in Korean population and examined these two SNPs. According to their results, only in smokers and individuals with squamous cell carcinoma does the association exist [4]. In spite of different races, Bae et al. arrived at part of the same conclusion as Wu et al. had. In addition, Xiao et al. used the meta-analysis firstly to collect and analyze a total of five case-control studies including 4608 lung cancer patients and 4617 case controls in Chinese population. They consider that SNP rs6495309 has an influence on the risk of lung cancer, and the allele C of SNP rs6495309 is a risk factor of lung cancer [10]. Based on the results above, I consider that SNP rs6495309 may have an impact on the risk of lung cancer. We did not arrive the related conclusion on account of insufficient samples in our study probably. From the proceeding discussion, I think whether SNP rs6495309 can affect the risk of lung cancer should be further studied. In addition, Jin et al. hold the opinion that the patients with CT/TT genotype give rise to better survival time compared with those carrying the homozygous wild genotype CC in Chinese Korean population [8]. This means that the allele T of SNP rs6495309 may be a protect factor of not only lung cancer but also survival time.

Nowadays, the morbidity and mortality of lung cancer stand first on the list of malignant tumor, and carcinogenic environment is an important factor of lung cancer. Our results indicate that there is a sharp positive interaction between polymorphism (rs6495309 rs1948 rs8040868) and smoking behaviors to the occurrence of lung cancer in Chinese Han population. Carcinogenic environment is not a determining factor, beyond that, individual susceptibility also plays an indispensable role in the occurrence and development of lung cancer. Our results support the view for the reason that SNP rs8040868 CC genotype has great effects on increasing the risk of lung cancer in nonsmokers as well as population less than or equal to 60 years old in our studies. More and more research shows that there is an interactive effect on lung cancer between genetic factors and environmental factors. And the nicotinic cholinergic receptor gene CHRNA5-CHRNA3-CHRNB4 located on 15q25 can influence the risk of lung cancer by affecting the nicotine dependence and downstream signaling pathways which contribute to increaser risk of lung cancer [37–40].

Tobacco and alcohol are the most commonly used drugs allover the world. Some studies have proved that CHRNA3 can have an effect upon smoking behaviors though high expression in key areas of the brain [41]. And individuals with CC genotype of SNP rs6495309 may consume more cigarettes, which leads to more damage to pulmonary function [41, 42]. Schlaepter et al. from the University of Colorado discovered that there is a link between SNP rs1948 and SNP rs8023462 on CHRNA5-CHRNA3-CHRNB4, and CC genotype of SNP rs1948 as well as TT genotype of SNP rs8023462 can influence people’s behaviors and obviously facilitate adults exposed to tobacco and alcohol earlier [7]. Nevertheless, Stephens et al. studied five SNPs on CHRNA5-CHRNA3-CHRNB4 in 56034 individuals from nine Caucasian countries including America and so on by Meta analysis. The results demonstrate that SNP rs1948, rs578776, rs588765 and rs684513 have protect effects on regular tobacco using but less influence on tobacco onset ages, while SNP rs16969968 is not significant for both of them. The results indicate that the cluster of genes CHRNA5-CHRNA3-CHRNB4 may exert different influences on early smoking behaviors and regular tobacco using [12].

In recent years, the incidence rate and fatality rate of lung cancer have increased significantly, many countries reported. They are the highest in the list of malignant tumors for male. And for female, lung cancer is the second common form of cancer and cause of cancer death. Non-small cell lung cancer (NSCLC) occupies 85 percent of lung cancer and this is the most frequency one [43]. The direct connection between polymorphism (rs6495309 rs8040868 rs1948) and squamous cell lung carcinoma or lung adenocarcinoma was not detected in our research. Some research also focused on different varieties of lung cancer. Some of the findings illustrated that there is an apparently relationship between SNP rs8040868 and the risk of non-small cell lung cancer in not only Chinese population but foreign population as well. Intriguingly, on one hand, Luo et al. accounted for that neither CHRNA3 SNP rs8040868 nor PHACTR2 SNP rs9390123 makes a difference on the risk of non-small cell lung cancer in Chinese population [5]. On the other hand, however, Chikova et al. appear to take a different view that CHRNA3 SNP rs8040868 as well as CHRNA9 SNP rs56159866 and rs6819385 give rise to a higher risk of non-small cell lung cancer, while the effect of CHRNA9 SNP rs55998310, rs56291234 and rs410759555 are exactly the opposite. And they also state that acetylcholine receptor α9 encoded by CHRNA9 gene and acetylcholine receptor α3 encoded by CHRNA3 gene paly a decisive role of lung caner susceptibility [11]. These mean that how SNP rs8040868 impact on the risk of non-small cell lung cancer may be different in Chinese and foreign population, and the factors of geography and races play the vital role in genetic polymorphism. Furthermore, eleven genome-wide association studies covering the population of China, America, Norway, Japan and Korea have discovered a few of susceptibility loci for lung cancer as well as chronic obstructive pulmonary disease. Yang et al. focused on CHRNA3 SNP rs6495309 and rs1051730 in 1511 COPD patients, 1559 lung cancer patients and 1677 case controls. Their study illustrated that individuals with CC/CT genotypes of SNP rs6495309 led to an increase in the risk of lung cancer and chronic obstructive pulmonary disease and poor prognosis. Moreover, there is a positive interaction between CC/CT genotypes and smoking behaviors to these diseases, while SNP rs1051730 was proved to have no effects on all on them [15]. However, we thought that there is no effects of SNP rs6495309, rs1948 and rs8040868 on the survival time of lung cancer and further research is necessary.

In addition, there are also plenty of studies that explore the influence of SNP rs6495309, rs1948 and rs8040868 on the other factors and fields. Marika et al. researched the relationship between 18 SNPs on CHRNA5-CHRNA3-CHRNB4 and body mass index (BMI) as well as blood pressure in the Northern Finland Birth Cohort 1966. Their results showed that the risk allele of SNP rs6495309, rs2036534, rs1996371, rs6495314, rs4887077 and rs11638372 had a negative impact on body mass index, and the allele G of SNP rs1948 brought out a lower average systolic blood pressure [2]. And Flora et al. applied the method of electrophoretic mobility shift assays (EMSAs) to probe whether three SNPs can make a difference to the gene expression of nucleoprotein cell line and small cell lung cancer (SCLC) cell lines. The results showed that the allele of SNP rs6495309 and rs8023462 could bind with nucleoprotein specifically, and the interaction between SNP rs8023462 and GATA transcription factor can impact on gene expression, while SNP rs1948 has on effect on them [1]. These studies indicate that there are still plenty of fields of the relationship between these three SNPs, rs6495309, rs1948 and rs8040868, and lung cancer deserve us to research and explore.

## MATERIALS AND METHODS

### Study subject

This was a hospital-based case-control study included 306 lung cancer patients and 306 cancer-free hospital controls. Among the lung cancer patients, there were 140 adenocarcinoma, 123 squamous cell carcinoma and 43 other types in pathologic type of lung cancer. All subjects were unrelated ethnic Han Chinese. There were no restriction of age and histology for the recruitment. The exclusion criteria of the cases included metastasized cancer, previous cancer and previous radiotherapy or chemotherapy. The cases were recruited at the First Affiliated Hospital of China Medical University. During the same time, controls were selected from cancer-free patients with other lung diseases, but who were free of a history of cancer, and mainly suffered from bronchitis and other pulmonary diseases. This study has been approved by China Medical University Ethics Committee.

### Data collection

A total of 2ml of venous blood was collected from each patient. Patients were interviewed face-to-face to collect information for demographics (name, gender, age, etc.), environmental exposure and smoking status by well-trained interviewers at the time that they were admitted to hospital. Information included dietary habit, demographic characteristics, family history of cancer, smoking exposure status and so on.

### Genotype analysis

Genomic DNA was extracted from peripheral blood samples using the standard phenol-chloroform extraction and ethanol precipitation. SNPs were genotyped by investigators blinded to case-control status in order to avoid any genotyping bias, using a TaqMan SNP genotyping assay (Affymetrix Inc., Cleveland, Ohio, USA) and read with the Sequence Detection Software on an Applied Biosystems 7500 FAST Real-Time PCR System (Foster City, CA, USA) using Each reaction (2ml) contained 5 μl TaqMan Genotyping master mix, 0.5 μl primers and probes (Applied Biosystems), 2.5 μl water and 2 μl DNA (15–25 ng/ml). Each plate included one negative control (no DNA). Thermal cycling was done under the following conditions: 95f for 10 min followed by 47 cycles of 92f for 30s and 60f for 1 min. Duplicates of 10% of the samples were selected and all duplicated were matched to validate the genotyping results.

### Statistical analysis

We used the Pearson’s chi square test to compare the differences between cases and controls. Unconditional logistic regression analysis was performed to calculate the odds ratios (OR) and their 95% confidence intervals (CI) for evaluating the associations between combination or interaction of SNPs and smoking factors with lung cancer. The Hardy-Weinberg equilibrium was tested by performing a χ^2^ test to compare the genotype frequencies of each SNP in the control subjects from those expected. All data were analyzed with Statistical Product and Service Solutions (SPSS) v13.0 for Windows, if not otherwise specified. All statistical analyses were two-sided and a P<0.05 was considered to be statistically significant.

## SUPPLEMENTARY MATERIALS TABLES



## Abbreviations

(CPD): cigarettes smoked per day
(GWAS): genome-wide association studies
(SNPs): single nucleotide polymorphisms
(nAChR): neuronal nicotinic acetylcholine receptor
(COPD): chronic obstructive pulmonary disease
(UTR): untranslated region
(TERT): telomerase reverse transcriptase
(MAF): minor allele frequency
(NSCLC): non-small cell lung cancer
(BMI): body mass index
(EMSAs): electrophoretic mobility shift assays
(SCLC): small cell lung cancer
(OR): odds ratios
(CI): confidence intervals
(SPSS): Statistical Product and Service Solutions
