# Supplementary material for: Study on polymorphisms in CHRNA5/CHRNA3/CHRNB4 gene cluster and the associated with the risk of non-small cell lung cancer
Source: Oncotarget. 2017 Dec 20;9(2):2435–44. doi: 10.18632/oncotarget.23459 (PMC5788651; doi:10.18632/oncotarget.23459)
Supplement: Supplementary file 1 [file oncotarget-09-2435-s001.pdf]

Study on polymorphisms in CHRNA5/CHRNA3/CHRNB4 gene cluster and the associated with the risk of non-small cell lung cancer

SUPPLEMENTARY MATERIALS

Supplementary Table 1: Associations between three SNPs and lung cancer risk, stratified by age

Age	Genotype	Cases(%)	Controls(%)	OR(95% CI)	*P* value	$OR_{adj.}$(95% CI)	$P_{adj.}$
<=60	rs6495309						
	CC	36(28.6)	34(24.1)	Ref	0.617	Ref	0.761
	CT	67(53.2)	76(53.9)	0.833(0.470-1.476)	0.53	1.089(0.579-2.048)	0.792
	TT	23(18.3)	31(22.0)	0.701(0.343-1.432)	0.329	0.833(0.378-1.836)	0.651
	CC+CT	103(81.7)	110(78.0)	Ref		Ref	
	TT	23(18.3)	31(22.0)	0.792(0.434-1.448)	0.449	0.788(0.402-1.546)	0.489
	rs8040868						
	TT	56(44.4)	68(49.3)	Ref	0.029	Ref	0.110
	TC	56(44.4)	67(48.6)	1.015(0.615-1.675)	0.954	0.984(0.566-1.711)	0.954
	CC	14(11.1)	3(02.2)	5.667(1.550-20.712)	0.009*	4.213(1.062-16.708)	0.041*
	TT+TC	112(88.9)	135(97.8)	Ref		Ref	
	CC	14(11.1)	3(02.2)	5.625(1.577-20.067)	0.008*	4.247(1.101-16.380)	0.036*
	rs1948						
	CC	24(19.2)	39(27.9)	Ref	0.258	Ref	0.165
	CT	74(59.2)	74(52.9)	1.625(0.890-2.967)	0.114	1.912(0.977-3.740)	0.058
	TT	27(21.6)	27(19.3)	1.625(0.778-3.396)	0.197	1.657(0.737-3.726)	0.222
	CC+CT	98(78.4)	113(80.7)	Ref		Ref	
	TT	27(21.6)	27(19.3)	1.153(0.634-2.097)	0.641	1.052(0.546-2.028)	0.879
>60	rs6495309						
	CC	54(30.2)	44(26.7)	Ref	0.736	Ref	0.756
	CT	86(48.0)	81(49.1)	0.865(0.524-1.427)	0.57	0.866(0.521-1.439)	0.579
	TT	39(21.8)	40(24.2)	0.794(0.439-1.439)	0.448	0.801(0.438-1.464)	0.471
	CC+CT	140(78.2)	125(75.8)	Ref		Ref	
	TT	39(21.8)	40(24.2)	0.871(0.527-1.439)	0.589	0.877(0.527-1.461)	0.614
	rs8040868						
	TT	68(38.0)	79(47.9)	Ref	0.181	Ref	0.240
	TC	92(51.4)	71(43.0)	1.505(0.962-2.357)	0.074	1.470(0.933-2.317)	0.097
	CC	19(10.6)	15(09.1)	1.472(0.695-3.117)	0.313	1.376(0.641-2.952)	0.412
	TT+TC	160(89.4)	150(90.9)	Ref		Ref	
	CC	19(10.6)	15(09.1)	1.188(0.582-2.422)	0.636	1.121(0.543-2.313)	0.757
	rs1948						
	CC	43(24.3)	52(31.7)	Ref	0.312	Ref	0.358
	CT	93(52.5)	77(47.0)	1.461(0.882-2.419)	0.141	1.430(0.857-2.386)	0.171
	TT	41(23.2)	35(21.3)	1.417(0.773-2.596)	0.26	1.408(0.761-2.605)	0.276
	CC+CT	136(76.8)	129(78.7)	Ref		Ref	
	TT	41(23.2)	35(21.3)	1.111(0.666-1.853)	0.686	1.120(0.666-1.883)	0.670

*$P<0.05$.

Supplementary Table 2: Associations between three SNPs and lung cancer risk, stratified by pathological subgroup

Pathological subgroup	Genotype	Controls(%)	No.(%)	OR(95% CI)	*P* value	$OR_{adj.}$(95% CI)	$P_{adj.}$
Adenocarcinoma	rs6495309						
	CC	78(25.5)	43(28.6)	Ref	0.642	Ref	0.812
	CT	157(51.3)	62(52.1)	0.891(0.541-1.469)	0.652	1.010(0.588-1.734)	0.972
	TT	71(23.2)	23(19.3)	0.743(0.400-1.380)	0.347	0.833(0.426-1.629)	0.594
	CC+CT	235(76.8)	95(80.7)	Ref		Ref	
	TT	71(23.2)	23(19.3)	0.801(0.473-1.358)	0.41	0.828(0.467-1.469)	0.519
	rs8040868						
	TT	147(48.5)	43(36.4)	Ref	0.04	Ref	0.238
	TC	138(45.5)	62(52.2)	1.536(0.976-2.416)	0.063	1.515(0.927-2.478)	0.098
	CC	18(5.9)	13(11.0)	2.469(1.120-5.441)	0.025*	1.449(0.635-3.304)	0.378
	TT+TC	285(94.1)	105(89.0)	Ref		Ref	
	CC	18(5.9)	13(11.0)	1.960(0.928-4.140)	0.078	1.157(0.531-2.519)	0.714
	rs1948						
	CC	91(29.9)	25(21.4)	Ref	0.137	Ref	0.190
	CT	151(49.7)	70(59.8)	1.687(0.998-2.854)	0.051	1.673(0.948-2.952)	0.076
	TT	62(20.4)	22(18.8)	1.292(0.669-2.493)	0.446	1.266(0.624-2.569)	0.513
	CC+CT	242(79.6)	95(81.2)	Ref		Ref	
	TT	62(20.4)	22(18.8)	0.904(0.526-1.553)	0.714	0.891(0.497-1.598)	0.698
Squamous	rs6495309						
cell carcinoma	CC	78(25.5)	39(28.9)	Ref	0.748	Ref	0.832
	CT	157(51.3)	67(49.6)	0.854(0.529-1.378)	0.517	0.908(0.558-1.476)	0.696
	TT	71(23.2)	29(21.5)	0.817(0.458-1.456)	0.493	0.836(0.466-1.500)	0.548
	CC+CT	235(76.8)	106(78.5)	Ref		Ref	
	TT	71(23.2)	29(21.5)	0.906(0.555-1.477)	0.691	0.890(0.542-1.460)	0.644
	rs8040868						
	TT	147(48.5)	61(45.2)	Ref	0.174	Ref	0.316
	TC	138(45.5)	59(43.7)	1.030(0.672-1.579)	0.891	1.029(0.668-1.584)	0.898
	CC	18(5.9)	15(11.1)	2.008(0.951-4.240)	0.067	1.778(0.833-3.792)	0.137
	TT+TC	285(94.1)	120(88.9)	Ref		Ref	
	CC	18(5.9)	15(11.1)	1.979(0.966-4.056)	0.062	1.753(0.847-3.628)	0.130
	rs1948						
	CC	91(29.9)	33(24.6)	Ref	0.318	Ref	0.318
	CT	151(49.7)	66(49.2)	1.205(0.737-1.972)	0.457	1.257(0.763-2.070)	0.369
	TT	62(20.4)	35(26.1)	1.557(0.876-2.766)	0.131	1.566(0.875-2.802)	0.131
	CC+CT	242(79.6)	99(73.9)	Ref		Ref	
	TT	62(20.4)	35(26.1)	1.380(0.857-2.221)	0.185	1.353(0.835-2.190)	0.219

*$P < 0.05$.

Supplementary Table 3: The relationship of the three SNPs and survival time

Genotype	Cases(%)	MST(month)	HR(95% CI)	*P* value	$HR_{adj.}$(95% CI)	$P_{adj.}$
rs6495309						
CC	90(29.4)	13	Ref	0.445	Ref	0.486
CT	153(50.0)	14.5	0.780(0.362-1.681)	0.526	0.768(0.356-1.658)	0.502
TT	63(20.6)	21	1.300(0.551-3.070)	0.549	1.242(0.520-2.968)	0.625
rs8040868						
TT	125(40.4)	22	Ref	0.291	Ref	0.290
TC	148(48.8)	14	1.539(0.743-3.189)	0.246	1.521(0.732-3.160)	0.261
CC	33(10.8)	13	2.088(0.782-5.573)	0.142	2.107(0.788-5.633)	0.137
rs1948						
CC	68(22.4)	39	Ref	0.23	Ref	0.214
CT	167(55.1)	12	1.179(0.492-2.827)	0.712	1.195(0.497-2.876)	0.691
TT	68(22.4)	13	2.033(0.797-5.186)	0.137	2.082(0.814-5.323)	0.126

MST: median survival time.
